# Bufalin down-regulates Axl expression to inhibit cell proliferation and induce apoptosis in non-small-cell lung cancer cells

**DOI:** 10.1042/BSR20193959

**Published:** 2020-04-09

**Authors:** Nam-Yi Kim, Young-Ah Suh, Soyoung Kim, ChuHee Lee

**Affiliations:** 1 Department of Pharmacology, School of Medicine, Dongguk University, Gyeongju 38066, South Korea; 2 College of Medicine, University of Ulsan, Asan Medical Center, Seoul 05505, South Korea; 3 Department of Biochemistry and Molecular Biology, School of Medicine, Yeungnam University, Daegu 42415, South Korea

**Keywords:** apoptosis, Axl, Bufalin, NSCLC

## Abstract

Axl, a member of the TAM (Tyro3, AXL, Mer) receptor tyrosine kinase family, plays critical roles in cell growth, proliferation, apoptosis, and migration. In the present study, we demonstrated that the anti-cancer activity of bufalin, a major bioactive component of the Chinese traditional medicine Chan Su, is mediated by the down-regulation of Axl in non-small-cell lung cancer (NSCLC) cells. We observed the inhibitory effect of bufalin on the proliferation of A549 and H460 NSCLC cells and the clonogenicity of these cells was reduced by bufalin treatment in a dose-dependent manner. Next, we found that the protein level of Axl was decreased in proportion to the concentration of bufalin in both A549 and H460 cells. Moreover, the promoter activity of the *Axl* gene was decreased by bufalin in a dose- and time-dependent manner, indicating that bufalin down-regulates *Axl* gene expression at the transcriptional level. We further examined if the anti-proliferative property of bufalin is influenced by Axl at the protein level. *Axl* overexpression attenuated the effect of bufalin in inhibiting cell proliferation and colony formation and inducing apoptosis in H460 cells, while knockdown of *Axl* gene expression induced the opposite effect. Taken together, our data indicate that the anti-proliferative and pro-apoptotic effects of bufalin were associated with the protein level of Axl, suggesting that Axl is a potent therapeutic target of bufalin in suppressing proliferation and inducing apoptosis in NSCLC cells.

## Introduction

Lung cancer is the most frequently diagnosed cancer in both men and women (14.5% and 8.4% of all diagnosed cases, respectively) and represents the leading cause of death in terms of mortality rate [1]. It is divided into two types that include small-cell lung carcinoma (15% of all cases) and non-small-cell lung carcinoma (NSCLC, 85%) [2]. Classic approaches such as surgery, adjuvant therapy, chemotherapy, and radiotherapy have been commonly used for treatment; however, customized therapy targeting appropriate oncogenes and immunotherapy have both been recently introduced. These novel strategies are considered as breakthrough treatments for improving the survival of NSCLC patients [3–7].

Axl, also known as Ark, Tyro7, or Ufo, is a receptor tyrosine kinase (RTK) that belongs to the TAM (Tyro3, Axl, MerTK) subfamily of RTKs [8,9]. Previous studies have demonstrated that Axl is overexpressed in a broad range of cancers and can transduce multiple signals that regulate cell growth, survival, proliferation, invasion, migration, angiogenesis [10], and apoptosis [11,12]. Axl is also reportedly correlated with highly aggressive cancers [13–15], epithelial-to-mesenchymal transition [16,17], and chemoresistance [17–19]. This protein is a practical therapeutic target for successful cancer treatment, and small molecules that inhibit Axl have been exploited. BGB324 (R248) is a selective Axl inhibitor and the first candidate to be tested in phase I clinical trials for treatment of acute myeloid leukemia or NSCLC [20,21]. BGB324-mediated Axl inhibition has been demonstrated to suppress the growth and metastasis of cancer cells and to induce apoptosis [21]. In addition to BGB324, a number of other inhibitors that function to suppress Axl expression and activation through different molecular mechanisms are under development [22].

Bufalin is one of the main active components of toad venom, which is a type of traditional Chinese medicine referred to as “Chan Su” or “Senso” [23]. Previous reports have demonstrated the anti-cancer effects of bufalin in the context of various types of cancers such as glioma [24], osteosarcoma [25], and breast [26], colorectal [27], ovarian [28], lung [29], pancreatic [30], gastric [31], and tongue cancer [32]. Bufalin is known to inhibit cell proliferation [24,28] and metastasis [31], induce apoptosis [32], and reverse acquired drug resistance [33,34]. Although the molecular mechanisms that underlie the anti-cancer activity of bufalin have been studied widely, bufalin’s ability to affect Axl expression and the outcome in response to bufalin treatment in this context remain unexplored.

In the present study, we demonstrated that bufalin inhibits cell proliferation and induces apoptosis in NSCLC cells, and these phenomena were found to be associated with the down-regulation of Axl expression. The findings suggest that Axl may act as a substantial therapeutic target of bufalin, contributing to the anti-cancer effects of this therapeutic agent.

## Materials and methods

### Reagents and antibodies

Bufalin was obtained from Sigma-Aldrich (St. Louis, MO, U.S.A.). A549 and H460 cells were purchased from the American Type Culture Collection (Manassas, VA, U.S.A.). Primers for Axl and glyceraldehyde 3-phosphate dehydrogenase (GAPDH) were synthesized by the domestic company, Bioneer Corp. (Daejeon, Korea). TRI reagent was obtained from Solgent Co., Ltd. (Daejeon, Korea). AmpliTaq DNA polymerase was obtained from Roche Diagnostics Corp. (Indianapolis, IN, U.S.A.). G418 was from Gibco BRL (Gaithersburg, MD, U.S.A.). Lipofectamine 2000 and mammalian expression vector, pcDNA3, were obtained from Invitrogen (Carlsbad, CA, U.S.A.). The plasmid, pGL3-basic vector, and the Dual-Glo luciferase assay kit were purchased from Promega Corp (Madison, WI, U.S.A.). Pre-validated Axl-targeting siRNA and control siRNA were purchased from Bioneer Corp. For Western blot analysis, specific antibodies against Axl, GAPDH, and secondary antibodies were obtained from Santa Cruz Biotechnology (Dallas, TX, U.S.A.).

### Cell culture

The A549 and H460 cells were grown in Roswell Park Memorial Institute (RPMI)-1640 medium (Gibco BRL) containing 10% fetal bovine serum (FBS), 2 mM L-glutamine, 10 U/ml penicillin, and 10 g/ml streptomycin at 37°C in 5% CO_2_ in a water-saturated atmosphere.

### Promoter activity measurement

The promoter reporter plasmid, pGL3-Axl, containing the *Axl* promoter region ranging from −556 to +7 bp of the transcriptional start site was prepared. Polymerase chain reaction (PCR) was carried out with 2 µl of genomic DNA and 1 µl of each primers (sense; 5′-GAAGGTACCAATGAAGGGCCAAGGAGGC-3′ and anti-sense; 5′-TTGGATCCGCACCGCCACGCCATGGGTG-3′). PCR conditions were 1 cycle of 3 min at 94°C, then 30 cycles of 30 s at 94°C, 30 s at 65°C, and 1 cycle of 5 min at 72°C. PCR-amplified DNA fragment was subcloned into the pGL3-basic vector, the promoterless luciferase plasmid. The constructed promoter–reporter plasmid was co-transfected into cells (3 × 10^5^ cells in a 60-mm dish) with renilla luciferase vectors, pRL-SV40, as an internal control. Luciferase activity was measured using a Dual-Glo luciferase assay system. According to the manufacturer’s instruction (Promega Corp, Madison, WI), luciferase assays were performed. Briefly, cell lysates were prepared from control cells as well as bufalin (20, 40 and 80 nM)-treated cells for 4 or 8 h using Passive Lysis Buffer. A 20 μl of cell lysates were mixed with 100 µl of firefly luciferase reagent (Luciferase Assay Reagent II) and then firefly luciferase activity (*Axl* promoter activity) was immediately measured. Next, 100 µl of Stop & Glo™ reagent was added to the reaction mixture and then *Renilla* luciferase activity was also measured. The ratio of firefly to Renilla luciferase activity was calculated.

### Western blot analysis

Total cell lysates were prepared from cells treated with the indicated concentrations (0, 20, 40 and 80 nM) of bufalin using lysis buffer [1% Triton X-100, 50 mM Tris (pH 8.0), 150 mM NaCl, 1 mM phenylmethylsulfonyl fluoride (PMSF), 1 mM Na_3_VO_4_, and protease inhibitor cocktail. Untreated cells were used as controls. Protein concentrations were determined using Bio-Rad protein assays. Proteins from the cell lysates (20–40 μg) were separated by 12% sodium dodecyl sulfate (SDS)-polyacrylamide gel electrophoresis (PAGE) and electrotransferred onto nitrocellulose membranes. The membranes were blocked for 30 min at room temperature in Tris-buffered saline with 0.05% Tween-20 (TTBS) containing 5% non-fat dry milk, and then incubated with TTBS containing a primary antibody for 4 h at room temperature. After three times of 10-min washes in TTBS, the membranes were incubated with peroxidase-conjugated secondary antibody for 1 h. Following three additional 10-min washes with TTBS, the protein bands of interest were visualized using an enhanced chemiluminescence detection system (Amersham™ ECL™ Prime Western Blotting Detection Reagent; GE Healthcare, Piscataway, NJ, U.S.A.). Density of each protein level was measured by LAS-3000 Fujifilm Image Reader and Multi-Gauge 3.0 software and Axl protein level was normalized with that of GAPDH.

### Reverse transcription PCR (RT-PCR)

Cells (2 × 10^5^) were seeded in a 60-mm culture dish and grown overnight and then treated with the indicated concentrations (0, 20, 40, 80 nM) of bufalin for 8 h. Total RNA was extracted using TRI reagent and subjected to cDNA synthesis and PCR. The specific primers were as follows: Axl sense, 5′-AACCTTCAACTCC TGCCTTCTCG-3′ and antisense, 5′-CAGCTTCTCCTTCAGC TCTTCAC-3′; GAPDH sense, 5′-GGAGCCAAAAGGGTCAT CAT-3′ and antisense, 5′-GTGATGGCATGGACTGTGGT-3′.

### Cell viability measurement

Cell viability was measured using Cell Counting Kit-8 assay kit (Dojindo Laboratories, Kumamoto, Japan). Cells (1 × 10^3^ cells/well) were seeded in 96-well plates and grown overnight and then treated with the indicated concentrations (0, 20, 40, or 80 nM) of bufalin for 24 or 48 h. At the end of treatment, 10 μl of CCK-8 solution was added and further incubated for 4 h. The absorbance at 570 nm was measured using a microplate reader (Model 680 microplate reader, Bio-Rad Laboratories). Values are normalized to that of untreated control cells to determine the % of viability and expressed as a percentage of the viable cells with respect to control cells.

### Colony formation assay

Cells were seeded into 24-well plates (1–2 × 10^3^ cells/well) and treated with the indicated concentrations (0, 20, 40, and 80 nM) of bufalin for 24 h and then washed with PBS to remove bufalin. Thereafter, cells were cultured for the next 7 to 10 days to form colonies. Colonies were stained with Crystal Violet (in 60% methanol; Junsei Chemical Co., Ltd., Tokyo, Japan) and images were acquired using the RAS-3000 Image Analysis System (FujiFilm, Tokyo, Japan). To quantify the numbers of colonies, Crystal Violet dyes were extracted from colonies using 10% acetic acid and the optical density of the resolved Crystal Violet dye was measured at 570 nm.

### Axl overexpression

To ectopically express Axl, the recombinant, pcDNA3-Axl, was constructed by cloning the Axl cDNA into the *Eco*RI and *Bam*HI sites of the pcDNA3 vector and 2 μg of purified plasmids were transfected into the A549 or A549/Cis cells (3 × 10^5^ cells in a 100-mm dish) using Lipofectamine 2000 (Invitrogen). To establish stable cell lines, which constitutively express Axl, the transfected cells were cultured in the presence of 400 μg/ml of G418. The RPMI 1640 medium containing G418 was refreshed every 3 days. After 3 to 4 weeks, the Axl-expressing cells were enriched and the Axl expression in these cells was analyzed by Western blot analysis.

### Small interfering RNA (siRNA) transfection

RNA interference-mediated gene silencing was performed to reduce Axl protein level. Cells (5 × 10^5^) were seeded in 60-mm culture dishes, grown overnight and then transfected with 100 nM siRNA targeting Axl (sense, 5′-AAGAUUUGGAGAdACACACUGA-3′ and antisense, 5′-UCAGUGUGUUCUCCAAAUCUU-3′), or control siRNA. The cells were harvested at 24 or 36 h after transfection and used to evaluate protein expression and cell proliferation, respectively.

### Detection of apoptosis

Fluorescence-activated cell sorting (FACS) analysis was performed to detect apoptotic cells. Cells (3 × 10^5^ cells in a 60-mm dish) were seeded, grown overnight, and then treated with 40 nM bufalin for 24 h. The harvested cells were washed once with ice-cold PBS and re-suspended in 100 μl of 1× binding buffer (HEPES buffered saline solution supplemented with 2.5 mM CaCl_2_) and then incubated with 2  µg/ml of Annexin V-fluorescein isothiocyanate (FITC) and 2.5 μg/ml of propidium iodide (PI) for 30 min at room temperature in the dark. Next, 900 µl of 1× binding buffer was added to the mixture and subjected to flow cytometry using a Becton-Dickinson FACS Caliber and analyzed by Cell Quest software (Becton-Dickinson, San Jose, CA, U.S.A.).

### Statistical analysis

Data were expressed as the means ± SD of triplicate samples or at least three independent experiments. To determine statistical significance, the Student’s *t*-test was used with a *P*-value threshold of <0.05.

## Results

### Bufalin inhibits the proliferation of NSCLC cells and induces apoptosis

**Figure 1:**
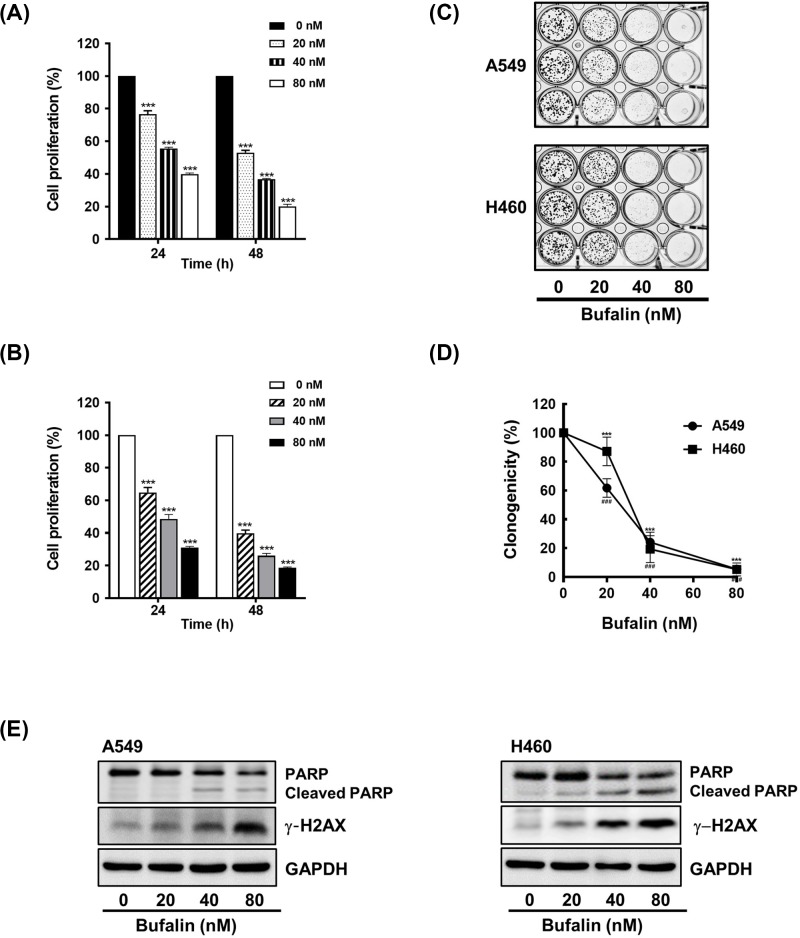
Bufalin inhibits cell proliferation and clonogenic activity and induces apoptosis (**A** and **B**) Cells were treated with 20, 40, or 80 nM bufalin for 24 or 48 h, and the viability of cells was measured using CCK-8 (***P<0.001 vs untreated group). (**C** and **D**) Cells (2 × 10^3^ cells/well) were exposed to 20, 40, or 80 nM bufalin for 24 h, washed with PBS, and then allowed to grow for the next 7 to 10 days. The colonies were visualized by Crystal Violet staining (*P*<0.001 (H460), ^###^*P*<0.001 (A549), vs untreated group). (**E**) Cells (3 × 10^5^ cells/ 60 mm dish) were exposed to 20, 40, or 80 nM bufalin for 24 h. The levels of PARP and γ-H2AX protein were determined by Western blot analysis.

We first examined the anti-proliferative effect of bufalin in the NSCLC cell lines A549 and H460. Cells were treated with 20, 40, or 80 nM concentration of bufalin for 24 or 48 h. As shown in Figure 1A,B, cell viability was reduced in response to bufalin treatment in a time- and dose-dependent manner. The IC_50_ values of bufalin after 24 h in A549 and H460 cells were 28.16 and 38.70 nM, respectively.

The inhibitory effect of bufalin on cell proliferation was further confirmed using colony formation assays. The clonogenic activity of cells treated with bufalin was decreased in proportion to the concentration of bufalin (Figure 1C,D). Of note, both A549 and H460 cells treated with 80 nM of bufalin were observed to be unable to form colonies.

Next, we assessed the effect of bufalin on apoptosis by examining apoptotic changes such as poly (ADP-ribose) polymerase (PARP) cleavage and the induction of γ-H2AX (the phosphorylation of H2AX). Western blot analyses revealed that the levels of PARP were decreased and that those of cleaved PARP were increased in response to bufalin. Moreover, a dose-dependent induction of γ-H2AX was also observed in both A549 and H460 cells that were treated with the indicated concentrations of bufalin (Figure 1E). Taken together, these results indicate that the anti-cancer effects of bufalin are a result of the inhibition of cell proliferation and the induction of apoptosis.

### Bufalin suppresses Axl expression at the transcriptional level

**Figure 2:**
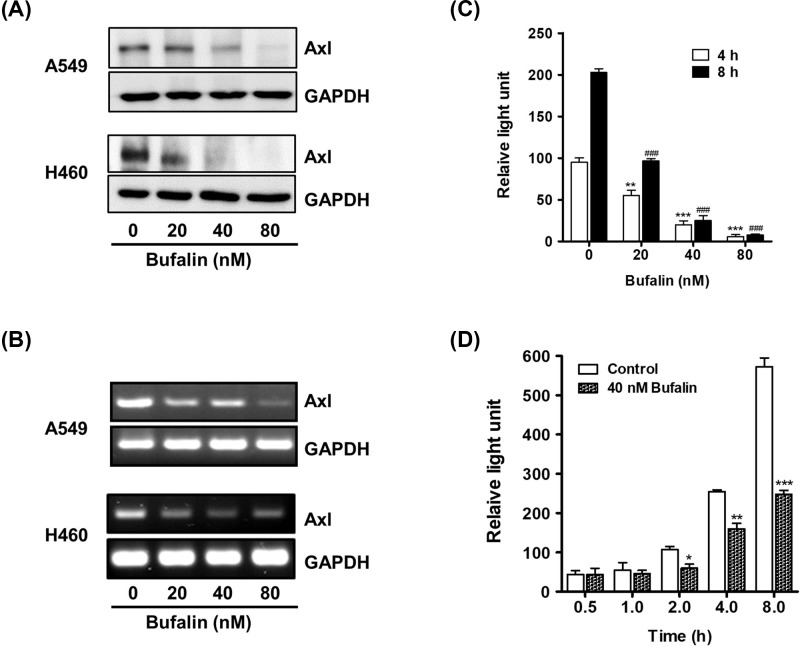
Bufalin reduces Axl protein level and suppresses promoter activity (**A**) Cells (3 × 10^5^ cells/ 60 mm dish) were treated with 20, 40, or 80 nM bufalin for 24 h. Axl protein levels were determined by Western blot analysis. (**B**) For RT-PCR, total RNAs extracted from the cells treated with the indicated concentrations of bufalin for 8 h were used to measure Axl mRNA levels. (**C**) To examine the effect of bufalin on Axl promoter activity, H460/pGL3-Axl cells (3 × 10^4^ cells) were incubated with 20, 40, or 80 nM bufalin for 4 or 8 h, and the luciferase activities were then measured (**P<0.01, ***P<0.001 (4 h), ^###^*P* < 0.001 (8 h), vs untreated group). (**D**) H460/pGL3-Axl cells were also treated with 40 nM bufalin for 0.5, 1, 2, 4, or 8 h, and then the luciferase activities were measured (*P<0.05, **P<0.01, ***P<0.001, vs untreated group).

As Axl is known to play critical roles in cell growth, proliferation, and survival, we investigated the effect of bufalin on Axl expression [35]. Western blot analyses revealed that bufalin treatment resulted in a dose-dependent decrease in the protein level of Axl in both A549 and H460 cells (Figure 2A). Notably, Axl protein expression was almost undetectable in H460 cells that were treated with 80 nM of bufalin.

The inhibition of Axl expression by bufalin treatment was also demonstrated using RT-PCR. Consistent with the results of Western blot, the mRNA levels of Axl in A549 and H460 cells were significantly decreased by bufalin treatment (Figure 2B). Additionally, the *Axl* promoter activity was assessed to further illustrate the effect of bufalin on *Axl* gene expression. Cells harboring *Axl* promoter-luciferase reporter plasmids were exposed to bufalin at the indicated concentrations for 4 or 8 h. The cells were also treated with 40 nM of bufalin for the indicated time periods. As shown in Figure 2C,D, bufalin treatment decreased luciferase activity in a dose- and time-dependent manner, indicating that bufalin suppressed the promoter activity of the *Axl* gene. Taken together, these results demonstrated that Axl expression was down-regulated by bufalin at the transcriptional level.

### The anti-proliferative effect of bufalin is attenuated or augmented in accordance with the protein level of Axl

**Figure 3:**
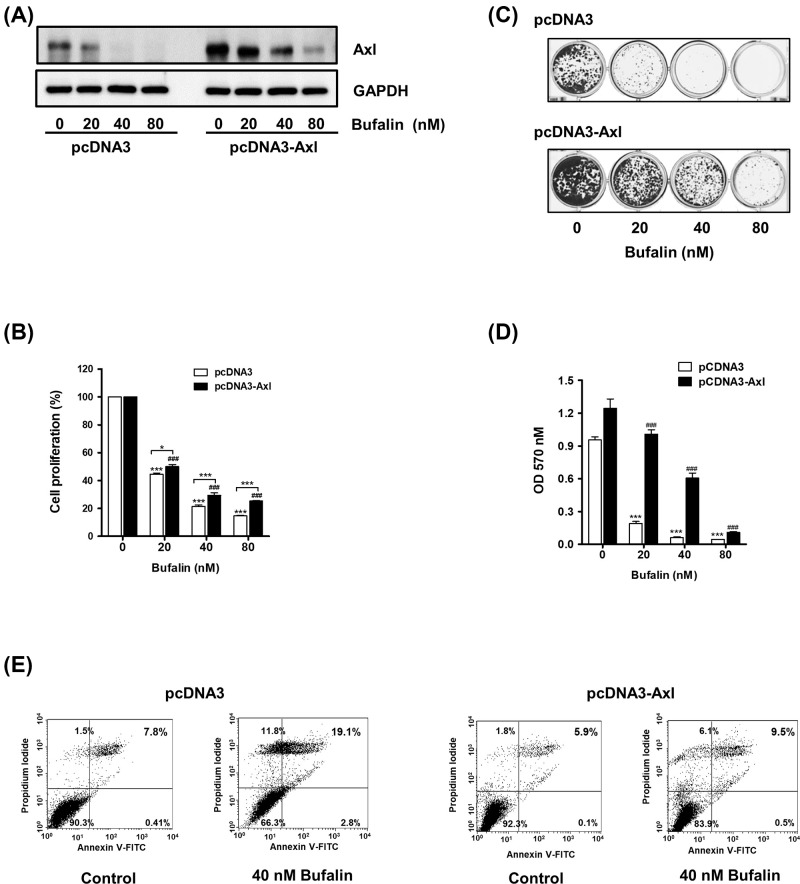
The inhibitory effects of bufalin on cell proliferation, colony formation, and induction of apoptosis are attenuated by Axl overexpression (**A**) H460/pcDNA3 or H460/pcDNA3-Axl cells (3 × 10^5^ cells/ 60-mm dish) were exposed to 20, 40, or 80 nM bufalin for 24 h, and Western blot analysis was conducted to determine Axl protein levels. (**B**) Cells (2 × 10^3^ cells/ 96 well) were treated with the indicated concentrations of bufalin for 24 h, and cell viability were measured using CCK-8. (***P<0.001 (H460/pcDNA3), ^###^*P*<0.001 (H460/pcDNA3-Axl), vs untreated group, *P<0.05, ***P<0.0001, H460/pcDNA3 vs H460/pcDNA3-Axl). (**C**) Cells (2 × 10^3^ cells/24 well) were treated with 20, 40, or 80 nM bufalin for 24 h, washed with PBS, and then allowed to grow for the next 7 to 10 days. The colonies were visualized by Crystal Violet staining. (**D**) For quantification of colony formation assay, colonies were destained and the absorbance at 570 nm was then measured. (***P<0.001, vs untreated group). (**E**) To detect apoptotic cells, cells were treated with 40 nM bufalin for 24 h, and the percentages of Annexin V and/or PI-stained cells were calculated.

We subsequently assessed if the inhibitory effect of bufalin on cell proliferation is related to the down-regulation of Axl expression. Axl-overexpressing cells (H460/pcDNA3-Axl)) were prepared via the transfection of recombinant plasmids encoding the *Axl* gene and then treated with 40 nM of bufalin (a median of the concentration range used in cell viability test for 24 h. Western blot analyses demonstrated that the protein level of Axl was higher in H460/pcDNA3-Axl cells than that in control H460/pcDNA3 cells that were transfected with the empty vectors (Figure 3A). As expected, even after 40 nM bufalin treatment, H460/pcDNA3-Axl cells possessed a higher protein level of Axl compared with that in H460/pcDNA3 cells. The proliferation of Axl-overexpressing cells was less affected by bufalin than that of control cells (Figure 3B). Additionally, Axl-overexpressing cells formed more colonies than did the control cells, although the overall clonogenic activity was reduced in proportion to the concentration of bufalin (Figure 3C,D). These results indicated that the anti-proliferative effect of bufalin is closely related to the protein level of Axl and can be attenuated by Axl overexpression, as demonstrated in H460/pcDNA3-Axl cells.

As Axl inhibition has been established to be linked to the induction of apoptosis [11,12,21], bufalin-induced apoptosis was further evaluated in both control and Axl-overexpressing cells via flow cytometry. As shown in Figure 3E, the percentage of early (Annexin V-positive and PI-negative, Annexin V^+^/PI^−^) and late apoptotic cells (positive for both Annexin V and PI, Annexin V^+^/PI^+^) was found to be increased from 0.41% and 7.8% in untreated control cells to 2.8% and 19.5% in bufalin-treated cells, respectively. However, in Axl-overexpressing cells, each population of early and late apoptotic cells increased from 0.1% and 5.9% in untreated cells to 0.5% and 9.5% in cells exposed to 40 nM bufalin, respectively. These results indicate that there is an inverse correlation between the protein level of Axl and the induction of apoptosis by bufalin in NSCLC cells.

**Figure 4:**
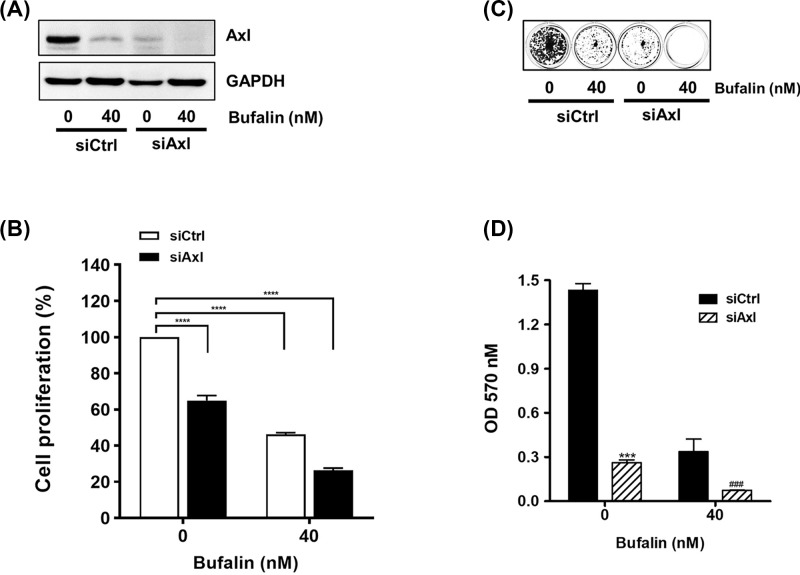
Knockdown of Axl expression augments the inhibitory effect of bufalin on cell proliferation and colony formation Cells (3 × 10^5^ cells/dish) transfected with control siRNA (H460/siCtrl)) or Axl-specific siRNA (H460/siAxl) were incubated with 40 nM bufalin for 24 to 36 h and then harvested. (**A**) The protein levels of Axl were determined by Western blot analysis. (**B**) Cells (2 × 10^3^ cells/96 well) were treated with 40 nM bufalin for 24 h, and then the viability of cells was measured using CCK-8. (****P<0.0001, vs untreated group). (**C** and **D**) Cells (2 × 10^3^ cells/24 well) were treated with 40 nM bufalin for 24 h, washed with PBS, and then allowed to grow for the next 7 to 10 days. For quantification, colonies were destained, and then the absorbance at 570 nm was measured (***P<0.001 (siCtrl),^ ###^*P*<0.001(siAxl), vs untreated cells).

We then examined the effect of an *Axl*-specific siRNA (siAxl) on the anti-proliferative activity of bufalin. As shown in Figure 4A, the protein level of Axl was decreased in H460/siAxl cells and was synergistically reduced by bufalin treatment. Consistent with the results of Western blot assays, inhibition of proliferation by bufalin was promoted in H460/siAxl cells to a higher degree than that in H460 cells transfected with control siRNA (Figure 4B). The clonogenicity of H460/siAxl cells exposed to bufalin was additionally decreased compared to that of H460/siCtrl cells (Figure 4C,D). Taken together, these results indicate that the protein level of Axl is proportional to the degree of cell proliferation, suggesting that Axl is a novel target of bufalin in interfering with the proliferation and clonogenic activity of NSCLC cells.

## Discussion

*Axl*, a proto-oncogene, was originally isolated from chronic myelogenous leukemia [8] and has been reported to be critical for survival, metastasis, angiogenesis, epithelial-to-mesenchymal transition, and drug resistance in many cancer cells, including NSCLC [5,10,16,18]. Accumulating evidence has demonstrated that interference with Axl expression and/or its activity results in inhibition of cell proliferation [36,37], angiogenesis [38], migration, and invasion in cancer cells [39,40], and results in a decrease in chemoresistance [41] and an increase in chemosensitivity [12,41] and the induction of apoptosis [11,12,20,21] in various cancer types. To date, Axl-targeting approaches, including small molecule kinase inhibitors [21,22], monoclonal antibodies [42–44], and decoy receptors [45], have been evaluated as potent anti-cancer therapies, and some of these approaches are currently under clinical development. Therefore, the importance of Axl as an anti-cancer target continues to receive more attention.

Bufalin is an active bufadienolide extracted from the skin and parotid venom glands of toads [46]. It has been reported to exhibit remarkable anti-cancer activity against a number of cancers such as gastric cancer [31], glioma [14], colorectal cancer [27], esophageal carcinoma [47,48], tongue cancer [32], osteocarcinoma [25], prostate cancer [49], and lung cancer [29]. In agreement with previous studies, the inhibitory effect of bufalin on cell proliferation was demonstrated in the NSCLC cell lines A549 and H460 (Figure 1A).

An increasing number of studies have revealed the molecular mechanisms responsible for the anti-cancer properties of bufalin. For example, it was found that bufalin treatment resulted in the down-regulation of Wnt/Achaete-scute-like 2 expression in gastric cancer [31], HIF-1α expression via inhibition of the phosphatidylinositol-4,5-bisphosphate 3-kinase/protein kinase B/mammalian target of rapamycin pathway in hepatocellular carcinoma [50], and heat shock protein-27 expression in osteosarcoma cells [51]. In the present study, NSCLC cells treated with bufalin exhibited reduced protein levels of Axl, a TAM RTK known to play important roles in cancer development, chemoresistance, and survival. Bufalin also suppressed the promoter activity of the *Axl* gene, indicating that it down-regulated Axl expression at the transcriptional level (Figure 2B,C). Additionally, bufalin-mediated anti-proliferative activity was attenuated or augmented by *Axl* gene overexpression or knockdown, respectively (Figures 3B–D and 4B–D). These results indicate that Axl inhibition by bufalin caused a significant reduction in cell proliferation, highlighting the inverse correlation between the protein expression of Axl and cell proliferation.

Multiple mechanisms are involved in bufalin-induced apoptosis. Bufalin has been shown to inactivate Na+/K+-ATPase in bladder carcinoma cells [52], reduce the expression of human telomerase reverse transcriptase in colorectal, pancreatic, and oral cancer cells [27,53], and down-regulate Mcl-1 expression in NSCLC cells [29,54]. It has also been shown to regulate apoptosis-associated proteins by increasing the expression of the anti-apoptotic protein B-cell lymphoma 2 and decreasing levels of the pro-apoptotic protein Bcl-2-associated X protein, apoptosis-inducing factors, and endonuclease G in tongue cancer cells [32]. Consistent with previous reports, in the present study, bufalin was also found to cause PARP cleavage and phosphorylation of H2AX, confirming the presence of bufalin-induced apoptosis in A549 and H460 cells (Figure 1E). Moreover, we found that bufalin-mediated apoptosis was less prominent in Axl-overexpressing cells than in control cells (Figure 3E), indicating that Axl is a target of bufalin that contributes to its pro-apoptotic activity.

In summary, we verified that bufalin down-regulates the expression of the *Axl* gene at the transcriptional level, in turn inhibiting cell proliferation and inducing apoptosis. Our data suggest that Axl is a valid therapeutic target for bufalin that is critical for its function as an anti-cancer agent in NSCLC cells.

## Abbreviations

Bcl-2: B-cell lymphoma 2 protein
NSCLC: non-small cell lung cancer
PARP: poly (ADP-ribose) polymerase
RTK: receptor tyrosine kinase
TTBS: Tris-buffered saline with 0.05% Tween-20

## References

[1] Bray F., Ferlay J., Soerjomataram I., Siegel R.L., Torre L.A. and Jemal A. (2018) Global cancer statistics 2018: GLOBOCAN estimates of incidence and mortality worldwide for 36 cancers in 185 countries. CA Cancer J. Clin. 68, 394–424 10.3322/caac.21492 30207593

[2] Chen Z., Fillmore C.M., Hammerman P.S., Kim C.F. and Wong K.K. (2014) Non-small-cell lung cancers: a heterogeneous set of diseases. Nat. Rev. Cancer 14, 535–546 10.1038/nrc3775 25056707 PMC5712844

[3] Peters S., Kerr K.M. and Stahel R. (2018) PD-1 blockade in advanced NSCLC: A focus on pembrolizumab. Cancer Treat. Rev. 62, 39–49 10.1016/j.ctrv.2017.10.002 29156447

[4] Chan B.A. and Hughes B.G. (2015) Targeted therapy for non-small cell lung cancer: current standards and the promise of the future. Transl. Lung Cancer Res. 4, 36–54 25806345 10.3978/j.issn.2218-6751.2014.05.01 PMC4367711

[5] Levin P.A., Brekken R.A., Byers L.A., Heymach J.V. and Gerber D.E. (2016) Axl Receptor Axis: A New Therapeutic Target in Lung Cancer. J. Thorac. Oncol. 11, 1357–1362 10.1016/j.jtho.2016.04.015 27130831 PMC4961571

[6] Gastonguay A., Berg T., Hauser A.D., Schuld N., Lorimer E. and Williams C.L. (2012) The role of Rac1 in the regulation of NF-kappaB activity, cell proliferation, and cell migration in non-small cell lung carcinoma. Cancer Biol. Ther. 13, 647–656 10.4161/cbt.20082 22549160 PMC3408971

[7] Chen Q.Y., Xu L.Q., Jiao D.M., Yao Q.H., Wang Y.Y., Hu H.Z. et al. (2011) Silencing of Rac1 modifies lung cancer cell migration, invasion and actin cytoskeleton rearrangements and enhances chemosensitivity to antitumor drugs. Int. J. Mol. Med. 28, 769–776 21837360 10.3892/ijmm.2011.775

[8] O'Bryan J.P., Frye R.A., Cogswell P.C., Neubauer A., Kitch B., Prokop C. et al. (1991) axl, a transforming gene isolated from primary human myeloid leukemia cells, encodes a novel receptor tyrosine kinase. Mol. Cell. Biol. 11, 5016–5031 10.1128/MCB.11.10.5016 1656220 PMC361494

[9] Lemke G. (2013) Biology of the TAM receptors. Cold Spring Harb. Perspect. Biol. 5, a009076 10.1101/cshperspect.a009076 24186067 PMC3809585

[10] Li Y., Ye X., Tan C., Hongo J.A., Zha J., Liu J. et al. (2009) Axl as a potential therapeutic target in cancer: role of Axl in tumor growth, metastasis and angiogenesis. Oncogene 28, 3442–3455 10.1038/onc.2009.212 19633687

[11] Cho C.Y., Huang J.S., Shiah S.G., Chung S.Y., Lay J.D., Yang Y.Y. et al. (2016) Negative feedback regulation of AXL by miR-34a modulates apoptosis in lung cancer cells. RNA 22, 303–315 10.1261/rna.052571.115 26667302 PMC4712679

[12] Linger R.M., Cohen R.A., Cummings C.T., Sather S., Migdall-Wilson J., Middleton D.H. et al. (2013) Mer or Axl receptor tyrosine kinase inhibition promotes apoptosis, blocks growth and enhances chemosensitivity of human non-small cell lung cancer. Oncogene 32, 3420–3431 10.1038/onc.2012.355 22890323 PMC3502700

[13] Gustafsson A., Martuszewska D., Johansson M., Ekman C., Hafizi S., Ljungberg B. et al. (2009) Differential expression of Axl and Gas6 in renal cell carcinoma reflecting tumor advancement and survival. Clin. Cancer Res. 15, 4742–4749 10.1158/1078-0432.CCR-08-2514 19567592

[14] Hutterer M., Knyazev P., Abate A., Reschke M., Maier H., Stefanova N. et al. (2008) Axl and growth arrest-specific gene 6 are frequently overexpressed in human gliomas and predict poor prognosis in patients with glioblastoma multiforme. Clin. Cancer Res. 14, 130–138 10.1158/1078-0432.CCR-07-0862 18172262

[15] Shieh Y.S., Lai C.Y., Kao Y.R., Shiah S.G., Chu Y.W., Lee H.S. et al. (2005) Expression of axl in lung adenocarcinoma and correlation with tumor progression. Neoplasia 7, 1058–1064 16354588 10.1593/neo.05640 PMC1501169

[16] Asiedu M.K., Beauchamp-Perez F.D., Ingle J.N., Behrens M.D., Radisky D.C. and Knutson K.L. (2014) AXL induces epithelial-to-mesenchymal transition and regulates the function of breast cancer stem cells. Oncogene 33, 1316–1324 10.1038/onc.2013.57 23474758 PMC3994701

[17] Byers L.A., Diao L., Wang J., Saintigny P., Girard L., Peyton M. et al. (2013) An epithelial-mesenchymal transition gene signature predicts resistance to EGFR and PI3K inhibitors and identifies Axl as a therapeutic target for overcoming EGFR inhibitor resistance. Clin. Cancer Res. 19, 279–290 10.1158/1078-0432.CCR-12-1558 23091115 PMC3567921

[18] Wu F., Li J., Jang C., Wang J. and Xiong J. (2014) The role of Axl in drug resistance and epithelial-to-mesenchymal transition of non-small cell lung carcinoma. Int. J. Clin. Exp. Pathol. 7, 6653–6661 25400744 PMC4230140

[19] Zhang Z., Lee J.C., Lin L., Olivas V., Au V., LaFramboise T. et al. (2012) Activation of the AXL kinase causes resistance to EGFR-targeted therapy in lung cancer. Nat. Genet. 44, 852–860 10.1038/ng.2330 22751098 PMC3408577

[20] Woo S.M., Min K.J., Seo S.U., Kim S., Kubatka P., Park J.W. et al. (2019) Axl Inhibitor R428 Enhances TRAIL-Mediated Apoptosis Through Downregulation of c-FLIP and Survivin Expression in Renal Carcinoma. Int. J. Mol. Sci. 20, 3253–3264 10.3390/ijms20133253 PMC6651098 31269715

[21] Chen F., Song Q. and Yu Q. (2018) Axl inhibitor R428 induces apoptosis of cancer cells by blocking lysosomal acidification and recycling independent of Axl inhibition. Am. J. Cancer Res. 8, 1466–1482 30210917 PMC6129480

[22] Rho J.K., Choi Y.J., Kim S.Y., Kim T.W., Choi E.K., Yoon S.J. et al. (2014) MET and AXL inhibitor NPS-1034 exerts efficacy against lung cancer cells resistant to EGFR kinase inhibitors because of MET or AXL activation. Cancer Res. 74, 253–262 10.1158/0008-5472.CAN-13-1103 24165158

[23] Datta P. and Dasgupta A. (2000) Interactions between drugs and Asian medicine: displacement of digitoxin from protein binding site by bufalin, the constituent of Chinese medicines Chan Su and Lu-Shen-Wan. Ther. Drug Monit. 22, 155–159 10.1097/00007691-200004000-00002 10774625

[24] Liu T., Wu C., Weng G., Zhao Z., He X., Fu C. et al. (2017) Bufalin Inhibits Cellular Proliferation and Cancer Stem Cell-Like Phenotypes via Upregulation of MiR-203 in Glioma. Cell. Physiol. Biochem. 44, 671–681 10.1159/000485279 29169175

[25] Lee C.H., Shih Y.L., Lee M.H., Au M.K., Chen Y.L., Lu H.F. et al. (2017) Bufalin Induces Apoptosis of Human Osteosarcoma U-2 OS Cells through Endoplasmic Reticulum Stress, Caspase- and Mitochondria-Dependent Signaling Pathways. Molecules 22 10.3390/molecules22030437 PMC6155407 28287444

[26] Li Y., Tian X., Liu X. and Gong P. (2018) Bufalin inhibits human breast cancer tumorigenesis by inducing cell death through the ROS-mediated RIP1/RIP3/PARP-1 pathways. Carcinogenesis 39, 700–707 10.1093/carcin/bgy039 29546393

[27] Zhang N., Xie Y., Tai Y., Gao Y., Guo W., Yu W. et al. (2016) Bufalin Inhibits hTERT Expression and Colorectal Cancer Cell Growth by Targeting CPSF4. Cell. Physiol. Biochem. 40, 1559–1569 10.1159/000453206 27997899

[28] Li H., Hu S., Pang Y., Li M., Chen L., Liu F. et al. (2018) Bufalin inhibits glycolysis-induced cell growth and proliferation through the suppression of Integrin beta2/FAK signaling pathway in ovarian cancer. Am. J. Cancer Res. 8, 1288–1296 30094101 PMC6079152

[29] Kang X.H., Zhang J.H., Zhang Q.Q., Cui Y.H., Wang Y., Kou W.Z. et al. (2017) Degradation of Mcl-1 through GSK-3beta Activation Regulates Apoptosis Induced by Bufalin in Non-Small Cell Lung Cancer H1975 Cells. Cell. Physiol. Biochem. 41, 2067–2076 10.1159/000475438 28419994

[30] Liu X., Xiao X.Y., Shou Q.Y., Yan J.F., Chen L., Fu H.Y. et al. (2016) Bufalin inhibits pancreatic cancer by inducing cell cycle arrest via the c-Myc/NF-kappaB pathway. J. Ethnopharmacol. 193, 538–545 10.1016/j.jep.2016.09.047 27686271

[31] Wang J., Cai H., Xia Y., Wang S., Xing L., Chen C. et al. (2018) Bufalin inhibits gastric cancer invasion and metastasis by down-regulating Wnt/ASCL2 expression. Oncotarget 9, 23320–23333 29805736 10.18632/oncotarget.24157 PMC5955089

[32] Chou H.Y., Chueh F.S., Ma Y.S., Wu R.S., Liao C.L., Chu Y.L. et al. (2017) Bufalin induced apoptosis in SCC4 human tongue cancer cells by decreasing Bcl2 and increasing Bax expression via the mitochondriadependent pathway. Mol. Med. Rep. 16, 7959–7966 10.3892/mmr.2017.7651 28983595 PMC5779878

[33] Sun J., Xu K., Qiu Y., Gao H., Xu J., Tang Q. et al. (2017) Bufalin reverses acquired drug resistance by inhibiting stemness in colorectal cancer cells. Oncol. Rep. 38, 1420–1430 10.3892/or.2017.5826 28731184 PMC5549034

[34] Yuan Z.T., Shi X.J., Yuan Y.X., Qiu Y.Y., Zou Y., Liu C. et al. (2017) Bufalin reverses ABCB1-mediated drug resistance in colorectal cancer. Oncotarget 8, 48012–48026 28624793 10.18632/oncotarget.18225 PMC5564622

[35] Braunger J., Schleithoff L., Schulz A.S., Kessler H., Lammers R., Ullrich A. et al. (1997) Intracellular signaling of the Ufo/Axl receptor tyrosine kinase is mediated mainly by a multi-substrate docking-site. Oncogene 14, 2619–2631 10.1038/sj.onc.1201123 9178760

[36] Li R., Shi X., Ling F., Wang C., Liu J., Wang W. et al. (2015) MiR-34a suppresses ovarian cancer proliferation and motility by targeting AXL. Tumour Biol. 36, 7277–7283 10.1007/s13277-015-3445-8 25895459

[37] Paccez J.D., Vasques G.J., Correa R.G., Vasconcellos J.F., Duncan K., Gu X. et al. (2013) The receptor tyrosine kinase Axl is an essential regulator of prostate cancer proliferation and tumor growth and represents a new therapeutic target. Oncogene 32, 689–698 10.1038/onc.2012.89 22410775 PMC4078100

[38] Tanaka M. and Siemann D.W. (2019) Axl signaling is an important mediator of tumor angiogenesis. Oncotarget 10, 2887–2898 10.18632/oncotarget.26882 31080559 PMC6499597

[39] Nam R.K., Benatar T., Wallis C.J.D., Kobylecky E., Amemiya Y., Sherman C. et al. (2019) MicroRNA-139 is a predictor of prostate cancer recurrence and inhibits growth and migration of prostate cancer cells through cell cycle arrest and targeting IGF1R and AXL. Prostate 79, 1422–1438 10.1002/pros.23871 31269290

[40] Uribe D.J., Mandell E.K., Watson A., Martinez J.D., Leighton J.A., Ghosh S. et al. (2017) The receptor tyrosine kinase AXL promotes migration and invasion in colorectal cancer. PLoS ONE 12, e0179979 10.1371/journal.pone.0179979 28727830 PMC5519024

[41] Palisoul M.L., Quinn J.M., Schepers E., Hagemann I.S., Guo L., Reger K. et al. (2017) Inhibition of the Receptor Tyrosine Kinase AXL Restores Paclitaxel Chemosensitivity in Uterine Serous Cancer. Mol. Cancer Ther. 16, 2881–2891 10.1158/1535-7163.MCT-17-0587 28904132 PMC5716844

[42] Duan Y., Luo L., Qiao C., Li X., Wang J., Liu H. et al. (2019) A novel human anti-AXL monoclonal antibody attenuates tumour cell migration. Scand. J. Immunol. 90, e12777 10.1111/sji.12777 31075180

[43] Koopman L.A., Terp M.G., Zom G.G., Janmaat M.L., Jacobsen K., Gresnigt-van den Heuvel E. et al. (2019) Enapotamab vedotin, an AXL-specific antibody-drug conjugate, shows preclinical antitumor activity in non-small cell lung cancer. JCI Insight 4, 11–19 10.1172/jci.insight.128199 31600169 PMC6948776

[44] Leconet W., Larbouret C., Chardes T., Thomas G., Neiveyans M., Busson M. et al. (2014) Preclinical validation of AXL receptor as a target for antibody-based pancreatic cancer immunotherapy. Oncogene 33, 5405–5414 10.1038/onc.2013.487 24240689 PMC5055582

[45] Kariolis M.S., Miao Y.R., Jones D.S. II, Kapur S., Mathews I.I., Giaccia A.J. et al. (2014) An engineered Axl ‘decoy receptor’ effectively silences the Gas6-Axl signaling axis. Nat. Chem. Biol. 10, 977–983 10.1038/nchembio.1636 25242553 PMC4372605

[46] Yang Q., Zhou X., Zhang M., Bi L., Miao S., Cao W. et al. (2015) Angel of human health: current research updates in toad medicine. Am. J. Transl. Res. 7, 1–14 25755824 PMC4346519

[47] Ding Y., Liu W., Wang X., Zhang L., Zhao M., Deng H. et al. (2018) Bufalin induces apoptosis in human esophageal carcinoma ECA109 cells by inhibiting the activation of the mTOR/p70S6K pathway. Oncol. Lett. 15, 9339–9346 29805658 10.3892/ol.2018.8526 PMC5958755

[48] Liu Y., Wang X., Jia Y. and Liu Y. (2017) Effects of bufalin on the mTOR/p70S6K pathway and apoptosis in esophageal squamous cell carcinoma in nude mice. Int. J. Mol. Med. 40, 357–366 10.3892/ijmm.2017.3039 28656204 PMC5504976

[49] Zhang J.J., Zhou X.H., Zhou Y., Wang Y.G., Qian B.Z., He A.N. et al. (2019) Bufalin suppresses the migration and invasion of prostate cancer cells through HOTAIR, the sponge of miR-520b. Acta Pharmacol. Sin. 40, 1228–1236 10.1038/s41401-019-0234-8 31028291 PMC6786369

[50] Wang H., Zhang C., Xu L., Zang K., Ning Z., Jiang F. et al. (2016) Bufalin suppresses hepatocellular carcinoma invasion and metastasis by targeting HIF-1alpha via the PI3K/AKT/mTOR pathway. Oncotarget 7, 20193–20208 26958938 10.18632/oncotarget.7935 PMC4991447

[51] Xie X.B., Yin J.Q., Wen L.L., Gao Z.H., Zou C.Y., Wang J. et al. (2012) Critical role of heat shock protein 27 in bufalin-induced apoptosis in human osteosarcomas: a proteomic-based research. PLoS One 7, e47375 10.1371/journal.pone.0047375 23091618 PMC3473020

[52] Li H., Wang P., Gao Y., Zhu X., Liu L., Cohen L. et al. (2011) Na+/K+-ATPase alpha3 mediates sensitivity of hepatocellular carcinoma cells to bufalin. Oncol. Rep. 25, 825–830 21181095 10.3892/or.2010.1120

[53] Tian X., Dai S., Sun J., Jiang S., Sui C., Meng F. et al. (2015) Bufalin Induces Mitochondria-Dependent Apoptosis in Pancreatic and Oral Cancer Cells by Downregulating hTERT Expression via Activation of the JNK/p38 Pathway. Evid. Based Complement. Alternat. Med. 2015, 546210 10.1155/2015/546210 26783410 PMC4689913

[54] Cao F., Gong Y.B., Kang X.H., Lu Z.H., Wang Y., Zhao K.L. et al. (2019) Degradation of MCL-1 by bufalin reverses acquired resistance to osimertinib in EGFR-mutant lung cancer. Toxicol. Appl. Pharmacol. 379, 114662 10.1016/j.taap.2019.114662 31301315

